# Pharmaceutical Marketing and the Invention of the Medical Consumer

**DOI:** 10.1371/journal.pmed.0030189

**Published:** 2006-04-11

**Authors:** Kalman Applbaum

## Abstract

Applbaum demystifies some of the assumptions at work in the "culture of marketing," toward the goal of explaining contemporary disease mongering.


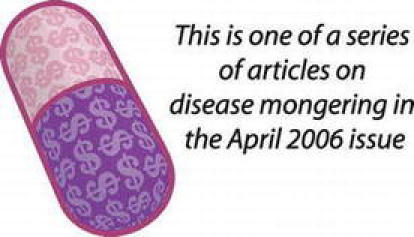


It is often said that leading drug companies now spend more on marketing than on research and development [ 1]. While such contemporary pharmaceutical marketing practices are sometimes believed to be a modern phenomenon, they are in fact a direct continuation of 19th-century patent medicine advertising. “Nostrum-mongers,” as the novelist Henry James dubbed them, are noted in the history of advertising as having been the leading spenders on, and foremost originators of, advertising technique [ 2, 3]. Nostrum sellers pioneered print advertising, use of trademarks and distinctive packaging, “pull” or demand-stimulation strategies, and even the design and commissioning of medical almanacs that functioned as vehicles for promotion of disease awareness. Henry James's psychologist brother, William James, was so exasperated by “the medical advertisement abomination” that in 1894 he declared that “the authors of these advertisements should be treated as public enemies and have no mercy shown” (see page 235 in [ 4]).

There is no doubt that drug company discoveries have profoundly improved upon our capacity to treat illness. But pharmaceutical marketing is more closely aligned with consumer marketing in other industries than with medicine, for which the consequences are not trivial. Once we view pharmaceutical industry activities in this light, we can disentangle industry's influence on contemporary medicine. Because we believe that we owe corporations our wealth and well-being, we tend not to question corporations' fundamental practices, and they become invisible to us. What follows is an attempt to demystify some of the assumptions at work in the “culture of marketing,” toward the goal of explaining contemporary disease mongering.

## Beliefs about the Free Market

There are three beliefs commonly associated with the “free market.” The first is that human beings are creatures of limitless but insatiable needs, wants, and discomforts. The second is that the free market is a place where these needs might be satisfied through the exercise of free choice. The last of these beliefs is that the surest avenue to innovation in all industries is unfettered competition in the market.

### Insatiable needs

The anthropologist Marshall Sahlins theorizes that the belief in unlimited wants is unique in the West, and stems from the Christian notion of “fallen man” as sufferer. This results, says Sahlins, in a peculiar idea of the person “as an imperfect creature of need and desire, whose whole earthly existence can be reduced to the pursuit of bodily pleasure and the avoidance of pain” [ 5]. A historical and philosophical examination of professional marketing shows that an assumption of boundless needs and wants is also at the heart of marketing theory. In this sense, marketing can be regarded as the institutionalization of this view of human nature. The marketer's challenge is to translate those limitless needs into profits.

Sahlins also points out that “in the world's richest societies, the subjective experience of lack increases in proportion to the objective output of wealth” [ 6]. In other words, the richer we get, the more we want. One explanation of this paradox lies in the way marketing activities are instrumental in getting us to think more about what we lack. Marketers and advertisers project and reflect back to us our discontent with the status quo. Americans are said to spend, on average, three years of their lives watching television advertisements, and the effect is that they are conditioned to want more and more. According to the advertisements, the viewer's personal anxieties and dissatisfactions are best addressed by consumption. This same message lies at the heart of much pharmaceutical advertising. [Fig pmed-0030189-g001]

**Figure pmed-0030189-g001:**
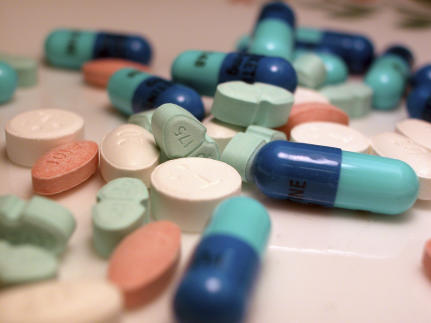
Pills are often marketed as a solution to human anxieties and dissatisfactions

### Lifestyle choices

In a consumer society, when individuals make choices toward the satisfaction of their needs and wants, they experience this as constructing their own individuality and identity. This special consumer identity is what people refer to when they use the word lifestyle, though they may not realize the consumerist implications of the word. Marketing claims to provide a solution to the problem of unlimited needs and wants, while simultaneously enhancing free choice and the construction of lifestyle.

In pharmaceuticals specifically, “lifestyle drug” marketing techniques were honed in the 1980s and 1990s for cosmetic and sexual enhancements [ 7, 8]. These techniques have been broadened to include other areas of medicine. The campaigns used to market cosmetic and sexual enhancements were focused on expanding perceived need for these products, and in this respect were a simple extension of customary marketing conduct that had existed for over half a century. The crossover to curative medicine occurred with psychotropic drugs, which have a very wide range of active properties, thus granting the marketer latitude in reinterpreting their value back to the consumer. For example, one class of antidepressants, the specific serotonin reuptake inhibitors, is marketed for eight distinct psychiatric conditions, ranging from social anxiety disorder to obsessive-compulsive disorder to premenstrual dysphoric disorder. And “lifestyle marketing” has now extended to the promotion of many of the blockbuster “maintenance drugs” intended for daily, lifelong consumption, such as drugs for allergies, insomnia, and acid reflux.

As a result of this sequence of events, industry opened the treatment of the inside of the body—the final frontier—to the same logic that governs all other marketing. Whether, in the antidepressant market, the “distribution channel captain,” as marketers refer to the predominant competitor, ends up sailing the serotonin reuptake channel (the serotonin reuptake inhibitors) or the norepinephrine reuptake channel (the challenger, serotonin–norepinephrine reuptake inhibitors) may yet be determined by marketing rather than by medical jockeying.

### Competition among drug companies yields innovation

It is an article of faith among free market devotees that breakthroughs spring not from paternalistic expert systems such as medicine but from industrial competition. As long as firms are committed to producing medications to treat diseases—as they are classified by medical science—this argument has some authority. But once a firm becomes principally driven by marketing—the case for most companies in most industries since the 1980s—then innovation comes to mean an elaboration of meaningless differences among a field of comparable “me too” products. “If marketing is seminally about anything,” said Theodore Levitt, one of the towering figures of marketing and former editor of the *Harvard Business Review*, “ it is about achieving customer-getting distinction by differentiating what you do and how you operate” [ 9]. More harmfully, expanding and altering the consumer's perception of disease is just as effective, and evidently a lot easier, than finding new cures.

An assumption of boundless needs and wants is at the heart of marketing theory.

## From Patients to Medical Consumers

Since, in a consumer society, we see ourselves as individuals and as free agents when we exercise consumer choice, it is not difficult for pharmaceutical companies and other privatized health-care deliverers to convince us that it is empowering to think of ourselves not as patients but as consumers. This conversion from patient to consumer also paves the way for the erosion of the doctor's role as expert. A startling report of this was described in a recent *New York Times* article: “For a sizable group of people in their 20's and 30's, deciding on their own what drugs to take—in particular, stimulants, antidepressants and other psychiatric medications—is becoming the norm. Confident of their abilities and often skeptical of psychiatrist's expertise, they choose to rely on their own research and each other's experience in treating problems like depression….A medical degree, in their view, is useful but not essential” [ 10]. This phenomenon, the article suggested, is “driven by familiarity” with the drugs. The emergence of this potentially dangerous situation demonstrates an unchecked expansion of the drug industry into an already accepted mode of thought—that “every minor mood fluctuation,” as the article reported, can and should be remedied.

Promoting consumer familiarity with drugs is one example of the very broad influence of the pharmaceutical industry. This influence extends to clinical trial administration, research publication, regulatory lobbying, physician and patient education, drug pricing, advertising and point-of-use promotion, pharmacy distribution, drug compliance, and the legal and ethical norms by which company practices themselves are to be evaluated. Actors traditionally found outside the “distribution channel” of the market are now incorporated into it as active proponents of exchange. Physicians, academic opinion leaders, patient advocacy groups and other grass roots movements, nongovernmental organizations, public health bodies, and even ethics overseers, through one means or another, have one by one been enlisted as vehicles in the distribution chain. The inclusion of patients in the distribution chain fundamentally changes their role from recipients of medical care to active consumers of the latest pharmaceuticals, a role which surely helps to support industry profits.

## Ethical Justification for Marketing

Because illness is one of the most tangible forms of suffering, the pharmaceutical industry, more than other industries, can link its marketing activities to ethical objectives. The result is a marriage of the profit-seeking scheme in which disease is regarded as “an opportunity” to the ethical view that mankind's health hangs in the balance. Marketers and consumers in the West to some extent share a common vision of needs and the terms of their satisfaction. This apparent complicity helps even the most aggressive marketers trust that they are performing a public service. Pharmaceutical company managers that I speak to signal this when they characterize their engagement with the public as “doing good while doing well.”

These managers also see nothing wrong with integrating doctors, patients, and other players into the drug distribution channel. On the contrary, they say, this is state-of-the-art management, making it professionally principled and tactically astute. Marketers also regard the incorporation of consumers into the channel as ethical because then people's needs can best be determined and satisfied, conferring upon them the power of self-determination through choice.

But this choice is an illusion. For in our pursuit of a near-utopian promise of perfect health, we have, without realizing it, given corporate marketers free reign to take control of the true instruments of our freedom: objectivity in science, ethics and fairness in health care, and the privilege to endow medicine with the autonomy to fulfill its oath to work for the benefit of the sick.
